# Mental Health of Immigrant Children and Adolescents (6–17 Years) in Canada: Evidence from the Canadian Health Measures Survey

**DOI:** 10.3390/ijerph20216997

**Published:** 2023-10-30

**Authors:** Oluwabukola Oladunni Salami, Maryna Yaskina, Katholiki Georgiades, Esperanza Diaz, Kathleen Hegadoren, Salima Meherali, Sophie Yohani, Ambikaipakan Senthilselvan

**Affiliations:** 1 Cumming School of Medicine, University of Calgary, Calgary, AB T2N 4Z6, Canada; 2 Women and Children’s Health Research Institute, University of Alberta, Edmonton, AB T6G 1C9, Canada; 3 Department of Psychiatry and Behavioural Neurosciences, McMaster University, Hamilton, ON L8N 3K7, Canada; georgik@mcmaster.ca; 4 Department of Global Public Health and Primary Care, University of Bergen, 5009 Bergen, Norway; 5 Faculty of Nursing, University of Alberta, Edmonton, AB T6G 1C9, Canada; kathy.hegadoren@ualberta.ca (K.H.); meherali@ualberta.ca (S.M.); 6 Department of Educational Psychology, University of Alberta, Edmonton, AB T6G 1C9, Canada; 7 School of Public Health, University of Alberta, Edmonton, AB T6G 1C9, Canada; sentil@ualberta.ca

**Keywords:** child, immigrant, mental health, Canada

## Abstract

Background: Studies indicate a higher prevalence of mental health problems among immigrants, but findings on immigrant children and adolescents are mixed. We sought to understand the magnitude of differences in mental health indicators between immigrant and non-immigrant children and adolescents in Canada and the influence of age, sex, household income, and household education. Methods: We completed a secondary analysis of data from the Canadian Health Measures Survey, using a pooled estimate method to combine data from four survey cycles. A weighted logistic regression was used to estimate the unadjusted and adjusted odds ratios with 95% confidence intervals. Results: We found an association between the mental health of immigrant versus non-immigrant children and adolescents (6–17 years) as it relates to emotional problems and hyperactivity. Immigrant children and adolescents had better outcomes with respect to emotional problems and hyperactivity/inattention compared to non-immigrant children and adolescents. Lower household socioeconomic status was associated with poorer mental health in children and adolescents. Conclusion: No significant differences in overall mental health status were evident between immigrant and non-immigrant children and adolescents in Canada but differences exist in emotional problems and hyperactivity. Sex has an influence on immigrant child mental health that varies depending on the specific mental health indicator.

## 1. Introduction

In 2021, Canada experienced significant milestones in its immigration landscape, highlighting its pivotal role in shaping the country’s demographic composition [1]. Notably, a historic 23.0% of the population consisted of current or former landed immigrants or permanent residents, a record high since Confederation [1]. This percentage surpassed even that of other G7 nations, such as France, Germany, Italy, Japan, the United Kingdom, and the United States [1]. This underscores the vital contribution of immigration in addressing the challenges posed by Canada’s aging population and declining birth rates [1]. From 2016 to 2021, Canada witnessed the arrival of over 1.3 million new permanent residents, marking the largest influx of recent immigrants ever recorded in a Canadian census [1]. Simultaneously, the proportion of second-generation Canadians, specifically children of immigrants under 15 years old with at least one foreign-born parent, increased from 26.7% in 2011 to 31.5% in 2021 [1]. These trends highlight the evolving dynamics of immigration and its impact on Canada’s demographic landscape.

Extensive research has underscored the considerable challenges surrounding mental health in immigrant populations [2,3,4]. Notably, studies examining mental health in these immigrant populations reveal a notable distinction between younger immigrants, including both children and adolescents, and their older counterparts [5,6,7]. Regarding the mental health of immigrant children and adolescents, the literature presents a diverse range of findings. Some investigations conducted in the context of Canada have reported a higher prevalence of mental health concerns among immigrant adolescent [8]. In another study among adolescents conducted within Canada, the authors reported a lower prevalence of these concerns [9]. Additionally, certain studies in Europe have not found a significant difference in mental health outcomes between immigrant and non-immigrant children and adolescents [10]. For example, a systematic review encompassing data from various European countries revealed that, in these diverse contexts, being an immigrant did not have an effect on emotional and behavioural problems in migrant children and adolescents [10]. On the other hand, refugee adolescents aged 12 to 19 were found to have an elevated prevalence of major depression and dysthymia compared to Québécois adolescents [8]. A global meta-analysis of 21 studies discovered that immigrants face elevated levels of mental health issues compared to non-immigrants [2]. Nonetheless, when it comes to the mental well-being of immigrant communities in Canada, the available information is inconclusive.

The disparities or commonalities in mental health outcomes between immigrant and non-immigrant children and adolescents can be understood through a multifaceted lens, drawing upon both theoretical frameworks and empirical evidence. This comprehensive approach is crucial for providing authors and readers with a clear understanding of why certain sociodemographic characteristics, such as sex, age, household income, and household education (including parental education), were chosen as focal points in our study. Acculturation theory posits that the process of adapting to a new culture can impact mental health outcomes among immigrants [11]. It suggests that the degree to which individuals maintain their cultural heritage while also integrating into the host culture can influence their psychological well-being. Additionally, the social determinants of the health framework emphasize that factors beyond access to healthcare, such as income, education, and social support, profoundly affect an individual′s mental health [12]. These determinants can vary between immigrant and non-immigrant populations and may contribute to mental health disparities. Empirically, previous research has consistently highlighted the importance of sociodemographic factors in shaping mental health outcomes among children and adolescents, including sex differences, age-related changes in emotional regulation, income disparities, and the role of parental education [13,14,15].

In addition, it is important to note that various social determinants of health, including age, income, sex, and education, have been implicated in immigrant health [6,7,9]. However, limited research has been conducted on the mental health of immigrant children below the age of 13 in Canada, including the prevalence of emotional and conduct problems. To address this gap in the literature, our study draws insights from the Canadian Health Measures Survey.

The purpose of this study was to examine the mental health of immigrant children and adolescents in Canada in comparison with non-immigrant children and adolescents while controlling for the influence of age, sex, household income, and household education.

## 2. Methods

We completed a secondary data analysis of the Canadian Health Measures Survey, a nationally representative biennial national survey that collects information about the health of Canadians through personal interviews and physical measures. [11] The survey collected data on participants aged 3 to 79 years in four cycles: 2007–2009, 2009–2011, 2012–2013, and 2014 to 2015.

The utilization of data from all four cycles of the Canadian Health Measures Survey was well-justified for several reasons. Firstly, it enhanced the study’s representativeness by providing a broader and more diverse sample. Secondly, it enabled the analysis of temporal trends in health measures, which was crucial for understanding how these measures changed over time. Additionally, the combination of data from these cycles yielded a substantial sample size, bolstering the statistical reliability of our analysis. Lastly, this approach allowed for a comprehensive examination of immigrant child mental health, including those who transitioned from refugee to permanent resident status, contributing to a more thorough understanding of this population’s health outcomes.

Each survey cycle involved data collection from around 5500 individuals, including immigrants and non-immigrants [16]. Immigrants were defined as individuals born outside of Canada. Only landed immigrants (permanent residents) were included in this study. Individuals who initially migrated as refugees but transitioned to permanent resident status were included in the sample. Our analysis focused on children and adolescents aged 6–17 years. Our final sample size was 7450 participants, of which 810 were immigrants.

Data were randomly collected from 16 communities across 10 provinces in Canada. Each cycle collects cross-sectional data on variables including country of birth, age, sex, household income, and household education. We selected these variables as studies have indicated these variables contribute to the mental health of immigrant children [6,7,9]. The Strengths and Difficulties Questionnaire (SDQ) was used to assess conduct problems, emotional problems, hyperactivity/ inattention, peer relationship problems, and prosocial behaviour [17]. While data were collected on individuals aged 3 to 79, data on child mental health using the SDQ were only available for children and adolescents aged 6 to 17 across all four cycles. The SDQ is a parent-reported behavioural screening questionnaire for children and adolescents ages 3 through 17. The tool has been proven reliable with a Cronbach’s alpha of 0.73 [17]. The survey has 25 questions, five of which relate to each mental health difficulty including conduct problems, emotional problems, hyperactivity/inattention, and peer relationship problems; the remaining five questions relate to prosocial behaviour—a measure of mental health strength. Each question is scored 0 (not true), 1 (somewhat true), or 2 (certainly true). Therefore, the maximum score that can be achieved for each domain is 10. Each domain is classified into normal, borderline, or abnormal as prescribed [18]. Of the five measures that were used as proxies for child mental health status, all (conduct problems, emotional problems, hyperactivity/ inattention, peer relationship problems) except prosocial behaviour were combined to achieve a total difficulties score.

Except for the prosocial behaviour score, a higher value indicates a greater mental health problem. In our analysis, conduct problems, emotional problems, hyperactivity/inattention, peer relationship problems, prosocial behaviour, and total difficulties scores were assessed as dichotomous variables (normal vs. borderline/abnormal). Sensitivity analyses were also carried out with the SDQ scores as nominal variables (normal vs. abnormal and borderline vs. abnormal) and the total difficulties score as a continuous variable.

### 2.1. Statistical Analysis

The data from the four Canadian Health Measures Survey (CHMS) cycles were appended according to the Statistics Canada guidelines and design weights. The design weights provided by Statistics Canada for the appended data were used to account for design effects and non-response bias and to ensure a representative sample for the Canadian population. Bootstrap weights provided by Statistics Canada were also used to obtain a standard error, level of significance, and 95 percent confidence interval in the logistic regression analysis. The outcome variables for the logistic regression analyses were the dichotomous variables corresponding to the outcome measures of interest. Odds ratios and 95% confidence intervals were used to describe the association between immigrant status and the outcomes after controlling for potential confounders. In the sensitivity analysis, we treated SDQ scores as nominal variables with three categories and employed multinomial logistic regression to assess the relationship between these SDQ scores and immigrant status while controlling for confounding factors. Additionally, we treated the total difficulties score as a continuous variable and utilized multiple regression analysis to examine the association between the total difficulties score and immigrant status while also controlling for confounding variables. Statistical analysis was performed using SAS software version 9.4 and STATA/MP software version 17.

### 2.2. Ethics Approval

We obtained ethics approval from the University of Alberta Research Ethics Board prior to data analysis.

## 3. Results

Characteristics of the study population are presented in Table 1.

### 3.1. Total Difficulties Score

Eighty-nine (89) percent of immigrant children had a normal total difficulties score compared to 87% of non-immigrant children (Table 2). No statistically significant (*p* > 0.05) difference was apparent in terms of the proportion of immigrant versus non-immigrant children who had a normal total difficulty score either in the unadjusted model or the model adjusting for age, sex, household income, and household education (adjusted OR 1.47; 95%CI 0.87–2.46) (Table 3). No significant association was observed between age and total difficulties score regardless of immigration status. Females were more likely to have a normal total difficulties score compared to males (Table 3). Children from households with an income of at least $80,000 were 2.2 times more likely to have a normal total difficulties score compared to children from households with an income less than $30,000 after adjusting for the other variables (adjusted: OR 2.19; 95%CI 1.46–3.29). Children from households whose highest level of education was secondary school were more likely to have a normal total difficulties score compared to those whose highest household education was less than secondary school (adjusted: OR 1.78; 95%CI 1.04–3.06). The significant association between being from a household whose highest level of education was post-secondary education and having a normal total difficulties score, as observed in the unadjusted model (OR 2.43; 95%CI 1.31–4.54), was attenuated in the presence of other variables in the model (OR 1.78; 95%CI 0.96–3.31).

### 3.2. Conduct Problems

Eighty-nine percent of immigrant children had a normal score in the conduct problems domain compared to 87% among non-immigrant children (Table 2). Unadjusted and adjusted models suggest no association (*p* > 0.05) between conduct problems and immigration status (unadjusted: OR 1.25; 95%CI 0.82–1.90; adjusted: OR 1.40; 95%CI 0.87–2.24) (Table 3).

No significant association between age and conduct problems was apparent (unadjusted: OR 1.04; 95%CI 0.99–1.08; adjusted: OR 1.04; 95%CI 0.99–1.08) regardless of immigration status. Females were more likely to have a normal score in the domain assessing conduct problems compared to males (unadjusted: OR 1.31; 95%CI 1.01–1.69; adjusted: OR 1.30; 95%CI 1.004–1.70).

Children from households with an income of at least $80,000 were over two times more likely to have a normal score in the domain that assessed conduct problems compared to children from households with an income less than $30,000 (unadjusted: OR 2.21; 95%CI 1.62–3.02; adjusted OR 2.09; 95%CI 1.44–3.02). Compared to children from a household with less than secondary school education, children from households with secondary education (unadjusted: OR 2.29; 95%CI 1.15–4.55; adjusted: OR 2.07; 95%CI 1.02–4.22) or post-secondary education (unadjusted: OR 2.65; 95%CI 1.46–4.82; adjusted: OR 1.96; 95%CI 1.02–3.79) were approximately two times more likely to have a normal score in the domain that assessed conduct problems (Table 3).

### 3.3. Emotional Problems

Eighty-six percent of immigrant children had a normal score in the domain that assessed emotional problems compared to 80% among non-immigrant children (Table 2). Immigrant children were significantly (*p* < 0.05) more likely to have a normal score in the domain that assessed emotional problems compared to non-immigrant children in both unadjusted and adjusted models (unadjusted: OR 1.52; 95%CI 1.12–2.08; adjusted: OR 1.78; 95%CI 1.30–2.44). Age was inversely associated with emotional problems among children in Canada regardless of their immigration status (unadjusted: OR 0.97; 95%CI 0.94–0.99; adjusted: OR 0.97; 95%CI 0.94–0.99). Females were less likely to have a normal score in the domain that assessed emotional problems compared to males (unadjusted: OR 0.65; 95%CI 0.53–0.80; adjusted: OR 0.68; 95%CI 0.54–0.85). No association was evident between household income and emotional problems among children. In the unadjusted model, children from households with secondary school (OR 1.82; 95%CI 1.11–2.96) or post-secondary education (OR 1.98; 95%CI 1.19–3.30) were statistically more likely to have a normal score in the domain that assessed emotional problems compared to children whose household highest level of education was less than secondary education. When adjusted by covariates, however, the observed associations were attenuated and became non-significant (Table 3).

### 3.4. Hyperactivity/Inattention

Eighty-eight percent of immigrant children had a normal score in hyperactivity/ inattention compared to 83% among non-immigrant children (Table 2). Both the unadjusted and adjusted models show immigrant children were significantly (*p* < 0.05) more likely to have a normal score in the domain that assessed hyperactivity/ inattention compared to non-immigrant children (unadjusted: OR 1.61; 95%CI 1.12–2.32; adjusted: OR 1.61; 95%CI 1.09–2.38). Age was positively associated with having a normal score for hyperactivity/ inattention (unadjusted: OR 1.06; 95%CI 1.02–1.09; adjusted: OR 1.06; 95%CI 1.02–1.10) regardless of immigration status. Females were significantly more likely to have a normal score in the domain that assessed hyperactivity/ inattention compared to males (unadjusted: OR 1.63; 95%CI 1.35–1.96; adjusted: OR 1.66; 95%CI 1.37–2.01). No association was found between household income and hyperactivity/ inattention in children except for households with an income of at least $80,000 compared to children from households with an income less than $30,000, but the results were not statistically significant in the adjusted model (unadjusted: OR 1.42; 95%CI 1.04–1.95; adjusted OR 1.30; 95%CI 0.92–1.82). No association was found between having a household education level of secondary school and hyperactivity/ inattention. However, we observed higher odds of having a normal score in the domain that assessed hyperactivity/ inattention among children whose household′s highest level of education was post-secondary compared to those whose household′s highest level of education was less than secondary school (unadjusted: OR 1.92; 95%CI 1.06–3.47; adjusted: OR 1.81; 95%CI 1.01–3.24) (Table 3).

### 3.5. Peer Relationship Problems

Eighty-seven percent of immigrant children had a normal score in the domain that assessed peer relationship problems compared to 86% among non-immigrant children (Table 2). Unadjusted and adjusted models suggest no association (*p* > 0.05) between peer relationship problems and immigration status (unadjusted: OR 1.12; 95%CI 0.74–1.69; adjusted: OR 1.22; 95%CI 0.78–1.92). No significant association was evident between age and peer relationship problems regardless of their immigration status. Similarly, we observed no significant association between sex and peer relationship problems. Children from households with an income of at least $80,000 were approximately two times more likely to have a normal score in the peer relationship problems domain compared to children from households with an income less than $30,000 (unadjusted: OR 1.87; 95%CI 1.36–2.57; adjusted OR 1.95; 95%CI 1.37–2.79). We observed no statistically significant associations between household education and peer relationship problems (Table 3).

### 3.6. Prosocial Behaviour

Ninety-seven percent of immigrant and non-immigrant children had a normal score in the domain that assessed prosocial behaviour (Table 2). No association (*p* > 0.05) was evident between immigration status and prosocial behaviour (unadjusted: OR 0.99; 95%CI 0.32–3.04; adjusted: OR 1.04; 95%CI 0.33–3.28) (Table 3), and no association was evident between age and prosocial behaviour among children in Canada regardless of their immigration status. Females were approximately four times more likely to have a normal score in the domain that assessed prosocial behaviour compared to males (unadjusted: OR 3.57; 95%CI 2.22–5.76; adjusted: OR 3.92; 95%CI 2.50–6.13). No associations were found between household income or household education and prosocial behaviour among children in Canada.

Sensitivity analyses were carried out with SDQ scores as nominal variables with three categories and the total difficulties score as a continuous variable. The results from the multinomial logistic regression comparing normal vs. abnormal were the same as those obtained from the logistic regression comparing normal vs. borderline/abnormal except for the associations between the total difficulties score and immigrant status being significant (RRR: 2.02; 95%CI: 1.10, 3.73). There was no significant association between SDQ scores and immigrant status when comparing borderline vs. abnormal except for the association between the total difficulties score and immigrant status (RRR: 1.89; 95%CI: 1.04, 3.43). When the total difficulties score was considered continuous in the multiple linear regression, there was a significant 1.14 unit reduction in the total difficulties scores for immigrants in comparison to non-immigrants (regression coefficient: −1.14; 95%CI: −1.85, −0.43; *p* = 0.002) indicating better mental health in immigrants than in non-immigrants.

## 4. Discussion

Our findings shed light on the complex landscape of mental health among children and adolescents in Canada. Notably, our study reveals that there were no significant differences in the overall mental health status between immigrant and non-immigrant children and adolescents aged 6 to 17 years. This trend in our data showing equal or even better mental health outcomes for immigrants compared to their non-immigrant counterparts aligns with previous research conducted in different parts of the world. For instance, in the Netherlands [19], a study reported that immigrant children′s mental health problems are on par with those of non-immigrant children. Similarly, in Hong Kong [20], migration has been found to have no significant correlation with overall self-esteem or depressive symptoms among children and adolescents. Furthermore, research conducted in Canada [21] has reported a statistically insignificant relationship between self-reported mental distress and immigration among children. These studies [19,20,21] collectively point to a consistent pattern of mental health parity or even advantages for immigrant children and adolescents compared to their non-immigrant peers. However, it′s important to acknowledge that there are nuances within the Canadian context. Research involving 203 adolescents aged 13 to 19 from 35 different countries in Quebec, Canada, has indicated a significant occurrence of mental health issues among immigrant adolescents [8], although this study did not compare immigrant and non-immigrant children and adolescents, but it suggests that mental health outcomes may not be uniform among immigrant youth and can vary based on specific factors or contexts. Conversely, a separate study conducted with Canadian immigrant adolescents has proposed a lower occurrence of mental health concerns [9]. This highlights the heterogeneity within immigrant populations and the need to consider various demographic and sociocultural factors when examining mental health outcomes. Additionally, a systematic review encompassing both immigrant and non-immigrant children and adolescents in Europe did not identify a significant disparity in mental health outcomes between these two groups [10]. This broader international perspective suggests that the relationship between immigration status and mental health is multifaceted and context-dependent. Furthermore, when we assessed each mental health domain individually, immigrant children and adolescents demonstrated better outcomes for emotional problems and hyperactivity/inattention. This finding aligns with earlier research [21], suggesting that immigrant adolescents tend to have lower levels of emotional–behavioural problems, further emphasizing the complexity of mental health outcomes within this population.

In our analysis, we also explored the relationships between various demographic factors and the mental health of children and adolescents, irrespective of their immigration status. These findings offer valuable insights into the nuanced interplay between demographics and mental well-being among youth. Firstly, we observed no significant association between age and overall mental health status, regardless of immigration status. However, as children and adolescents grew older, a noteworthy trend emerged: the likelihood of experiencing emotional problems increased, while the prevalence of hyperactivity/inattention decreased. This underscores the importance of considering developmental factors when assessing and addressing mental health in young people [22]. Regarding gender differences, our study revealed that female children and adolescents fared better than their male counterparts across multiple dimensions of mental health, including overall mental health status, conduct problems, hyperactivity/inattention, and prosocial behaviour. These findings align with the results reported by Georgiades et al. [23] in the Ontario Child Health Study, where a higher prevalence of parent-reported mental health disorders was observed among male compared to female children and adolescents. These gender-related variations underscore the need for gender-sensitive approaches to support the unique mental health challenges faced by male children and adolescents.

Furthermore, our analysis affirmed a well-established link between socioeconomic status and mental health. This association, documented in previous studies [7,24,25], was corroborated by our findings. Specifically, children and adolescents from households with higher income or education levels exhibited better mental health status compared to their counterparts from lower-income or lower-education households. These results emphasize the significant impact of socioeconomic factors on mental well-being and emphasize the importance of addressing disparities in access to resources and opportunities to promote better mental health outcomes, particularly among disadvantaged children and adolescents.

This study boasts several notable strengths that enhance the credibility and significance of its findings. Firstly, mental health assessment relied on a validated questionnaire, contributing to the robustness and reliability of the reported outcomes. The use of a well-established tool for measuring mental health not only ensures consistency but also allows for meaningful comparisons with other studies. Secondly, the study′s strength lies in its utilization of a representative nationwide sample, as opposed to a more limited province-based approach seen in some previous research focused on the impact of immigration status on child mental health. This broader scope provides a more comprehensive and holistic understanding of the mental health landscape among children and adolescents across Canada. Lastly, the study′s approach of assessing multiple individual measures of mental health status is noteworthy. This methodological choice allowed for the identification of nuanced differences in the prevalence of emotional problems and hyperactivity/inattention between immigrant and non-immigrant children and adolescents, even though there were no discernible differences in overall mental health status.

Despite these strengths, several important caveats should be considered when interpreting the study’s results. One notable limitation is the potential for self-report bias, which is inherent to questionnaires reliant on subjective responses. Individuals may have a tendency to over-report favourable outcomes and under-report less favourable ones, a phenomenon well-documented in psychological research. This introduces a degree of subjectivity into the data, and researchers must be cautious in interpreting self-reported mental health assessments. Another limitation arises from the inability to conduct sub-group analyses, particularly based on race and ethnicity, within the immigrant population due to insufficient sample size. This limitation prevents a more granular examination of differences related to immigration routes or country of origin. For example, mental health outcomes may differ significantly between refugees and those who migrated through other avenues, such as federal skilled workers. Further exploration of these nuances could provide valuable insights into the mental health disparities within the immigrant population.

Moreover, the study′s design precluded the possibility of conducting interaction analyses to assess potential effect modifications for other variables by immigration category. This limitation limits our ability to fully understand how various demographic and socioeconomic factors interact with immigration status to influence mental health outcomes among children and adolescents. Lastly, it is important to acknowledge that mental health is a culturally sensitive concept, and questions regarding the cultural sensitivity of the Strengths and Difficulties Questionnaire (SDQ) have been raised [17,18]. However, the study asserts that the SDQ has demonstrated validity and reliability across diverse populations and immigrant groups. Nevertheless, the potential influence of cultural factors on mental health assessments should be recognized when interpreting the results [26,27].

## 5. Conclusions and Further Studies

This study implies that immigrant children and adolescents in Canada may experience lower levels of emotional problems and hyperactivity compared to their non-immigrant counterparts. However, it is important to note that the influence of gender on the mental health of immigrant children varies across specific mental health indicators. To gain a more comprehensive understanding of the mental health outcomes among immigrant children and adolescents in Canada, further research is warranted. Such studies should employ validated screening and assessment tools and encompass diverse immigrant groups.

Additionally, interventions designed to support immigrant youth can prioritize addressing health disparities that may be influenced by both gender and income. By recognizing these nuances, healthcare and social services can better cater to the unique mental health needs of immigrant children and adolescents in Canada. In future studies, it is crucial to emphasize that while our current research suggests that the mental health status of immigrant children and adolescents in Canada may not significantly differ from that of non-immigrant children and adolescents, there is a critical need for more targeted investigations.

Specifically, studies should aim to provide deeper insights into the experiences of subgroups, including vulnerable populations such as refugee children and adolescents, as well as the children and adolescents of temporary residents, such as students and workers. Given their often more traumatic experiences and challenges in securing employment or dealing with limited income due to study-related restrictions, it is vital to explore whether their children may face worse mental health outcomes compared to more traditional migrant (permanent resident) children and adolescents considered in this study.

Furthermore, future research should thoroughly address the question of cultural sensitivity concerning the Strengths and Difficulties Questionnaire (SDQ) to assess its effectiveness in capturing the mental health of immigrant children from diverse regions of the world, such as South Asia. Lastly, it is imperative to stress the necessity for studies that effectively examine potential differences related to race/ethnicity and regional factors, as these factors can significantly impact the mental health outcomes of immigrant children and adolescents in Canada.

## Figures and Tables

**Table 1:** Characteristics of immigrant versus non-immigrant children and adolescents.

Characteristics	Immigrants N *(%) 810 (11.6)	Non-Immigrants N *(%) 6650 (88.4)
Sex		
Male	400 (50)	3400 (52)
Female	400 (50)	3250 (48)
Household Income (in Canadian Dollars)		
$0–$29,999	150 (20.5)	700 (10.8)
$30,000–$49,999	150 (17.7)	900 (12.7)
$50,000–$79,999	200 (26.1)	1500 (24.2)
$80,000 and more	300 (35.6)	3500 (52.3)
Household Education		
Less than secondary school graduation	50 (3.5)	200 (3.4)
Secondary school graduation	50 (10.1)	900 (14.5)
Post-secondary graduation	700 (86.4)	5200 (82.1)
Age at interview, Mean (SD)	12.1 (0.2)	11.6 (0.04)

* Due to Statistics Canada policy, raw frequencies were rounded to the nearest 50.

**Table 2:** Comparison of outcome variables for immigrant versus non-immigrant children and adolescents.

SDQ Subscales	Immigrants N *(%)		Non-Immigrants N *(%)		Immigrants 95%CI		Non-Immigrants 95%CI		Significance Level
	Normal	Not Normal	Normal	Not Normal	Normal	Not Normal	Normal	Not Normal	
Total difficulties score	650 (89.3)	100 (10.7)	5450 (86.6)	800 (13.4)	84.8–93.7	6.3–15.2	85.1–88.1	11.9–14.9	NS
Conduct problems	650 (89.1)	100 (10.9)	5400 (86.8)	800 (13.2)	84.9–93.3	6.7–15.1	85.6–87.9	12.0–14.4	NS
Emotional problems	600 (85.7)	100 (14.3)	5000 (79.7)	1200 (20.3)	82.3–89.1	10.9–17.7	77.9–81.5	18.5–22.1	S
Hyperactivity/ inattention	650 (88.4)	100 (11.6)	5150 (82.6)	1050 (17.4)	84.7–92.1	7.9–15.3	80.9–84.2	15.8–19.1	S
Peer relationship problems	650 (86.8)	100 (13.2)	5350 (85.5)	900 (14.5)	82.6–91.1	8.9–17.4	83.9–87.1	12.9–16.0	NS
Pro-social behaviour	700 (96.8)	<50 (3.2)	6050 (96.8)	200 (3.2)	94.4–99.3	0.72–5.6	96.1–97.6	2.4–3.9	NS

* Due to Statistics Canada policy, raw frequencies were rounded to the nearest 50; NS = No statistically significant difference at *p* < 0.05; S = Statistically significant difference at *p* < 0.05.

**Table 3:** Odds ratios and 95% confidence intervals for total difficulties score, conduct problems, emotional problems, hyperactivity/ inattention, peer relationship problems, and pro-social behaviour.

Characteristics	Unadjusted Model	Adjusted Model
	OR (95%CI)	OR (95%CI)
**Total Difficulties Score**		
*Migration status*		
Non-immigrant	Reference	Reference
Immigrant	0.78 (0.48, 1.27) 1.28 (0.79, 2.03)	1.47 (0.87, 2.46)
*Age (y)*	1.03 (0.99, 1.07)	1.03 (0.99, 1.07)
*Sex*		
Male	Reference	Reference
Female	1.21 (0.98, 1.51)	1.33 (1.04, 1.69)
*Household Income*		
$0–$29,999	Reference	Reference
$30,000–$49,999	0.97 (0.65, 1.47)	0.98 (0.65, 1.49)
$50,000–$79,999	1.23 (0.82, 1.85)	1.18 (0.77, 1.80)
$80,000 and more	2.26 (1.51, 3.37)	2.19 (1.46, 3.29)
*Household Education*		
Less than secondary school	Reference	Reference
Secondary school graduation	1.96 (1.14, 3.38)	1.78 (1.04, 3.06)
Post-secondary graduation	2.43 (1.31, 4.54)	1.78 (0.96, 3.31)
**Conduct Problems**		
*Migration status*		
Non-immigrant	Reference	Reference
Immigrant	1.25 (0.82, 1.90)	1.40 (0.87, 2.24)
*Age (y)*	1.04 (0.99, 1.08)	1.04 (0.99, 1.08)
*Sex*		
Male	Reference	Reference
Female	1.31 (1.01, 1.69)	1.30 (1.004, 1.70)
*Household Income*		
$0–$29,999	Reference	Reference
$30,000–$49,999	0.98 (0.65, 1.48)	0.93 (0.62, 1.40)
$50,000–$79,999	1.45 (0.99, 2.12)	1.46 (0.97, 2.18)
$80,000 and more	2.21 (1.62, 3.02)	2.09 (1.44, 3.02)
*Household Education*		
Less than secondary school	Reference	Reference
Secondary school graduation	2.29 (1.15, 4.55)	2.07 (1.02, 4.22)
Post-secondary graduation	2.65 (1.46, 4.82)	1.96 (1.02, 3.79)
**Emotional Problems**		
*Migration status*		
Non-immigrant	Reference	Reference
Immigrant	1.52 (1.12, 2.08)	1.78 (1.30, 2.44)
*Age (y)*	0.97 (0.94, 0.99)	0.97 (0.94, 0.99)
*Sex*		
Male	Reference	Reference
Female	0.65 (0.53, 0.80)	0.68 (0.54, 0.85)
*Household Income*		
$0–$29,999	Reference	Reference
$30,000–$49,999	0.87 (0.61, 1.25)	0.84 (0.56, 1.24)
$50,000–$79,999	1.19 (0.87, 1.62)	1.17 (0.85, 1.63)
$80,000 and more	1.43 (0.99, 2.06)	1.41 (0.98, 2.03)
*Household Education*		
Less than secondary school	Reference	Reference
Secondary school graduation	1.82 (1.11, 2.96)	1.69 (0.999, 2.85)
Post-secondary graduation	1.98 (1.19, 3.30)	1.63 (0.98, 2.73)
**Hyperactivity/Inattention**		
*Migration status*		
Non-immigrant	Reference	Reference
Immigrant	1.61 (1.12, 2.32)	1.61 (1.09, 2.38)
*Age (y)*	1.06 (1.02, 1.09)	1.06 (1.02, 1.10)
*Sex*		
Male	Reference	Reference
Female	1.63 (1.35, 1.96)	1.66 (1.37, 2.01)
*Household Income*		
$0–$29,999	Reference	Reference
$30,000–$49,999	1.03 (0.70, 1.53)	1.00 (0.66, 1.52)
$50,000–$79,999	0.94 (0.67, 1.32)	0.88 (0.62, 1.26)
$80,000 and more	1.42 (1.04, 1.95)	1.30 (0.92, 1.82)
*Household Education*		
Less than secondary school	Reference	Reference
Secondary school graduation	1.42 (0.80, 2.52)	1.46 (0.82, 2.60)
Post-secondary graduation	1.92 (1.06, 3.47)	1.81 (1.01, 3.24)
**Peer Relationship Problems**		
*Migration status*		
Non-immigrant	Reference	Reference
Immigrant	1.12 (0.74–1.69)	1.22 (0.78, 1.92)
*Age (y)*	0.96 (0.92, 1.01)	0.96 (0.92, 1.003)
*Sex*		
Male	Reference	Reference
Female	1.16 (0.90, 1.48)	1.26 (0.97, 1.64)
*Household Income*		
$0–$29,999	Reference	Reference
$30,000–$49,999	1.06 (0.68, 1.66)	1.13 (0.73, 1.75)
$50,000–$79,999	1.29 (0.88, 1.89)	1.34 (0.89, 2.02)
$80,000 and more	1.87 (1.36, 2.57)	1.95 (1.37, 2.79)
*Household Education*		
Less than secondary school	Reference	Reference
Secondary school graduation	1.28 (0.74, 2.23)	1.17 (0.67, 2.04)
Post-secondary graduation	1.76 (1.001, 3.08)	1.34 (0.74, 2.41)
**Pro-social Behaviour**		
*Migration status*		
Non-immigrant	Reference	Reference
Immigrant	0.99 0.32, 3.04)	1.04 (0.33, 3.28)
*Age (y)*	0.97 (0.89, 1.05)	0.97 (0.90, 1.04)
*Sex*		
Male	Reference	Reference
Female	3.57 (2.22, 5.76)	3.92 (2.50, 6.13)
*Household Income*		
$0–$29,999	Reference	Reference
$30,000–$49,999	2.05 (0.62, 6.81)	2.28 (0.62, 8.43)
$50,000–$79,999	1.62 (0.60, 4.34)	1.70 (0.59, 4.87)
$80,000 and more	1.88 (0.82, 4.30)	2.01 (0.80, 5.09)
*Household Education*		
Less than secondary school	Reference	Reference
Secondary school graduation	2.41 (0.64, 8.97)	2.37 (0.68, 8.32)
Post-secondary graduation	2.04 (0.58, 7.18)	1.72 (0.48, 6.27)

Odds ratios from bivariable and multivariable logistic regressions for having “Normal” scores.

## Data Availability

Data for this study is available by contacting Statistics Canada. This article is based on a secondary analysis of the Canadian Health Measures Survey. Access to data from the Canadian Health Measures Survey is available through Statistics Canada.

## Abbreviations

CI—confidence interval; CHMS—Canadian Health Measures Survey: OR—odds ratio; RRR—relative risk ratio; SDQ—Strengths and Difficulties Questionnaire.
